# Detection of viable oral bacteria of the patient on the surgical mask of dentists

**DOI:** 10.1038/s41405-023-00182-4

**Published:** 2024-01-16

**Authors:** Madline Priska Gund, Jusef Naim, Janina Lang, Matthias Hannig, Barbara Gärtner, Alexander Halfmann, Gabor Boros, Stefan Rupf

**Affiliations:** 1 Department of Operative Dentistry, Periodontology and Preventive Dentistry, Saarland University, Homburg, Germany; 2 Oral Surgery Clinic, German Armed Forces Central Hospital, Koblenz, Germany; 3 Institute of Medical Microbiology and Hygiene, Department of Hospital Hygiene, Saarland University, Homburg, Germany; 4 Synoptic Dentistry, Saarland University, Homburg, Germany

**Keywords:** Infection control in dentistry

## Abstract

**Introduction and aim:**

Bioaerosols contaminate the personal protective equipment (PPE), especially masks. The PPE harbors microorganisms from various sources. However, no previous studies have investigated the specific sources of bacteria found on used masks and their correlation with those from the treated patient.

**Setting, design, material and methods:**

Intraoral samples from the patient were collected prior to dental aerosol-producing treatments using a nylon flock fiber swab. After treatment, the practitioner’s mask was imprinted onto agar plates.

**Main outcome methods:**

Following cultivation, colony forming units were counted and identified using matrix-assisted laser desorption/ionization time-of-flight mass spectrometry (MALDI-TOF MS). After the samples were analyzed, the intraoral samples as well as the mask samples were assessed for the presence of identical species, which were subsequently quantified.

**Results:**

126 treatments were included. One species match occurred most frequently (26.2%), followed by two (11.9%%) and three or more (3.97%). In the intraoral samples, *Neisseria subflava* occurred most often, within mask samples *Staphylococcus epidermidis* were detected most. *Staphylococcus aureus* could be cultivated three times more often in intraoral samples than on the mask.

**Discussion and conclusion:**

Oral microorganisms originating from the patient’s oral cavity can be found on the outside of masks. When using PPE during treatments, it should therefore always be in mind that potentially pathogenic microorganisms may land on the mask becoming a source of for itself.

## Introduction

Wearing personal protective equipment (PPE) has been given great importance in dentistry, and not just since the 2019 coronavirus (Covid-19) outbreak.

Aerosol-producing dental treatments generate bioaerosols contaminating the practitioner, assistant, patients and environment. Standard PPE is therefore recommended for every dental treatment preventing contamination, transmission and possible infection with or by pathogens. This includes gloves, face masks and protective goggles. Further precautions are advisable (e.g. protective gown, face shield).

Contamination of PPE has already been intensively investigated [1–5]. Especially the bacterial contamination rate of the mask [6–9], with and without a protective face shield [10] was examined. Moreover, one research group was able to demonstrate that the mask itself has a contamination potential after aerosol-producing treatments [11, 12].

It is assumed that bacteria from aerosols originate from the patient (calculus, biofilm, blood, saliva) [13–15]. However, also patient-independent sources such as contaminated water pipes and general air contamination are known factors [16, 17].

So far, it has never been demonstrated from which source the bacteria on the used mask originate. In order to prevent PPE contamination as much as possible, it is important to know and assess the sources correctly.

The aim of this study was therefore to investigate the sources of contamination, starting with the mask by evaluating replication competent bacteria from the mask and the corresponding patient.

## Material and methods

### Setting

This prospective study was conducted at Saarland University Hospital, Clinic of Operative Dentistry, Periodontology and Preventive Dentistry. All instruments used for treatment were sterile. The treatment unit and surrounding surfaces were routinely disinfected (using Celtex® Wipes, Lotfex, Bremen, Germany; Incidin® 0,25%, Dräger, Lübeck, Germany). The room temperature was 20–22 °C, the relative humidity 40–60%.

### Patients

Only adult patients without known infectious diseases were included in the study. Use of antibiotics in the last 6 months resulted in exclusion from the study. All samples were anonymized. Both verbal and written informed consent were obtained from all participants. Ethical approval for the study was obtained (Vote No. 195/22).

### Subjects

Experiments were performed by 14 specially instructed and supervised second-year clinical dental students. Hygienic hand disinfection was performed before donning PPE. The surgical mask was picked up by the ties while wearing gloves, the assistant knotted it behind the head. All participants were instructed not to touch the exterior surface of their surgical mask. After treatment, masks were removed by the assistant. PPE consisted of pathogenfree (“non-sterile”) medical gloves (nitrile powder-free gloves: Joza®, Hebei Titans Hongsen Medical Technology Co., Ltd., Hebei, China), surgical masks (tie-band medical surgical mask type II, Mölnlycke Health Care, Düsseldorf, Germany), protective eyewear (Safeview® eyewear, Halyard, Neunkirchen, Germany), hair caps (BARRIER® Nurses Cap, Mölnlycke Health Care AB, Göteborg, Schweden und FarStar® medical GmbH, Barsbüttel, Germany) and protective gown (Simani Industrie s.r.l., Gallicano, Italy). Sampling was conducted from October 2022 to February 2023.

### Treatment

126 aerosol-producing treatments were included: professional tooth cleanings, periodontal treatments, filling therapies and endodontic treatments. For all types of treatments, water from the dental unit was used for cooling. An evacuation was established by means of conventional dental suction (CDS) using a cannula of 3.3 mm in diameter (suction flow 1.1 l/s) and a high-volume evacuation (HVE) of 8.0 mm in diameter (suction flow 6.0 l/s). The CDS was placed lingual to the lower central incisors. The HVE was held near the aerosol source by an assistant. Professional tooth cleaning included the supragingival cleaning with ultrasonics and manual instruments of the entire dentition and afterwards polishing. Periodontal cleaning included the subgingival cleaning with ultrasonics and manual instruments of all diseased pockets. Filling therapy and endodontic treatment was mainly performed with a rubber dam. The caries excavation was performed without a rubber dam. The filling was placed with a rubber dam if it was not subgingival. It was removed again for finishing. Trepanation was performed without a rubber dam in order to properly assess the axis of the tooth. All further steps were performed under rubber dam. In each case, one tooth was treated, rarely several.

### Sampling

Prior to treatment, intraoral samples were taken with a nylon flock fiber swab (eSwab™universal, Mast Diagnostica, Reinfeld, Germany). For this purpose, the swab was passed retromolar on the terminal lower right molar along the mucogingival border of the lingual surface of the front teeth and buccally back. The swab was then stored in Amies medium.

After the treatment, the practitioner´s surgical mask was removed by the assistant without touching the exterior surface of the mask. It was then imprinted immediately onto two different agar plates: Columbia (Columbia III Agar with 5% sheep blood, Becton Dickinson GmbH, Heidelberg, Germany) and Chocolate agar (GC II Agar with Hemoglobin and IsoVitaleX; Becton Dickinson GmbH, Heidelberg, Germany) plates for 10 s each.

### Microbial cultivation

In order to cultivate the intraoral sample, 100 μl of the Amies medium was transferred to each agar plate using the triple-streak plating method. For this purpose, bacteria were removed from the bacterial suspension using an inoculation loop (Sarstedt, Nümbrecht, Germany) and the first zigzag streak was made on the agar plate. Bacterial density was then reduced by passing a second sterile inoculation loop through the first streak- but only two to three times. This procedure was repeated using a third sterile inoculation loop to further reduce bacterial density and facilitate isolation of different species for subsequent analysis. The Columbia agar plates were placed in incubation containers for gas generating systems (AnaeroPack Rectangular Jar, Mitsubishi Gas Chemical Company, ING., Tokyo, Japan). A gas bag (GasPak CO2 Container System, Becton Dickinson GmbH, Heidelberg, Germany) to enrich a carbon dioxide rich environment was inserted and the container was sealed and incubated at 35° +/− 2° for 48 h.

### Quantitative bacterial analysis

Colony forming units were counted using a colony counter (schuett-biotec GmbH, Göttingen, Germany).

### Qualitative bacterial analysis

All phenotypically distinguishable colony forming units grown on the agar plates were classified using matrix-assisted laser desorption/ionization time-of-flight mass spectrometry (MALDI-TOF Biotyper^TM^ MBT^TM^ smart, Bruker Daltonik GmbH, Bremen, Germany). Colonies were picked and transferred to a stainless-steel target (MSP 96 spot target, Bruker Daltonik GmbH, Bremen, Germany) and overlayed with 1 μl of formic acid (AppliChem GmbH, Darmstadt, Germany). After it dried, 1 μl of matrix (Bruker HCCA = α-Cyano-4-hydroxycinnamic acid, Bruker Daltonik GmbH, Bremen, Germany) was applied. Measurements were continued until the bacterium was clearly identified. If this was not the case, the measurement was carried out again. If a spectrum could still not be assigned to a known species, it was noted as “unidentified”.

### Matching species

After the samples were analyzed, the intraoral samples as well as the mask samples were assessed for the presence of identical species, which were subsequently quantified. The concordance was documented regarding a single species, two species, and three or more species.

### Controls

**Fig. 1:**
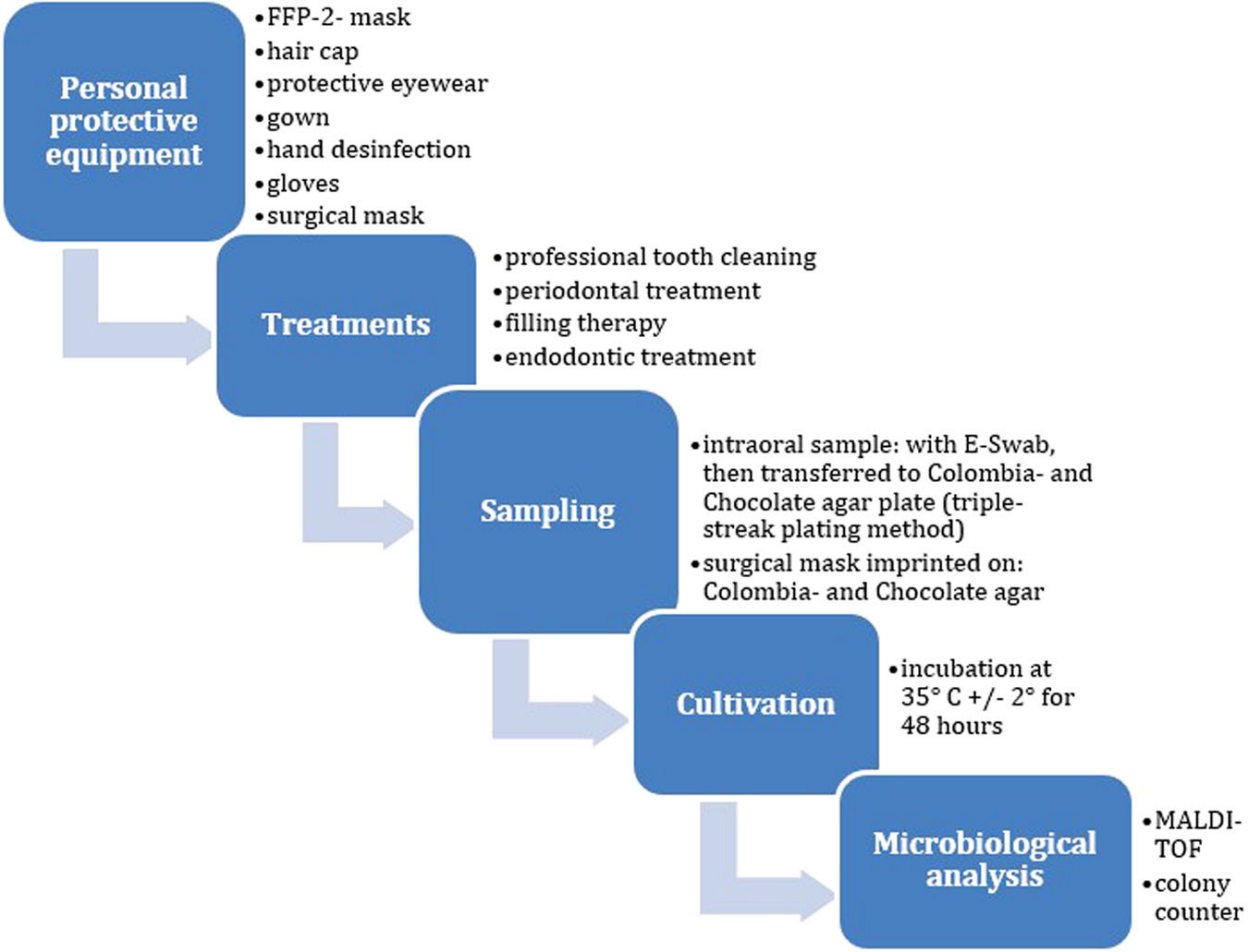
Flow chart. Flow chart of the study.

Five unused surgical masks (*n* = 5) were worn for 120 min each during simulated aerosol-producing dental treatments (trepanation, cavity preparation) on a phantom simulator and used as negative controls. No intraoral swab was taken, as it was a phantom head (Fig. 1).

### Statistics

Qualitative and quantitative results of bacteria in intraoral samples and bacterial contamination of surgical masks were presented descriptively. Frequencies of detection of bacteria in intraoral samples and surgical masks overall as well as for the treatment modalities were compared using the One-Way ANOVA for repeated measures. For the comparison of the detection frequencies of bacteria between different treatment modalities from intraoral samples and from surgical masks, the One-Way ANOVA for independent measures was used (*p* < 0.05).

## Results

### Controls

After 48 h of cultivation, no bacterial growth was observed in any of the control samples.

### Quantitative results

126 aerosol-producing treatments were included: 50 professional tooth cleanings, 33 periodontal treatments, 31 filling therapies and 12 endodontic treatments. The average duration of treatment was 120 min.

**Fig. 2:**
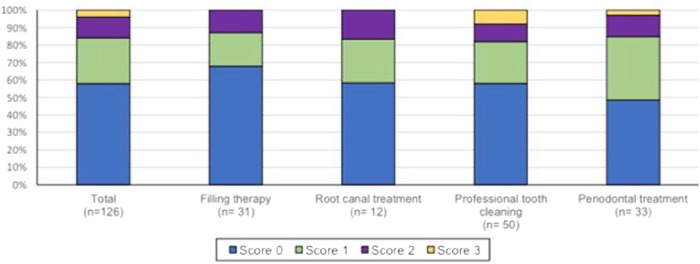
Number matching species (comparison of bacteria of mask and intraoral sample) in percent according to treatment modality.

One matching species occurred most frequently (in total: 26.2%), followed by two matching species (in total: 11.9%). The rarest, three matching species or more were found (in total: 3.97%). There were only minor differences depending on the treatment modality. Three matching species or more could not be determined for filling therapy and endodontic therapy (Fig. 2).

### Qualitative results

The identified microorganisms are presented in Supplementary Table 1. *S. epidermidis* was found most frequently, followed by *N. subflava, M. luteus, A. oris*. Concerning the intraoral samples, most often *N. subflava* occurred, followed by *A. oris, H. parainfluenzae, R. dentocariosa*. With regard to the mask samples *S. epidermidis, M. luteus, S. hominis* and *S. capitis* could be detected most frequently in descending order. *S. aureus* was cultivated three times more often in the intraoral samples than on the mask. It was most frequently detected before filling therapy (4 times) and professional tooth cleaning (4), but also before periodontal (1) and endodontic treatment (1). On the mask, *S. aureus* could only be cultivated after professional tooth cleaning (3).

### Statistical analysis

Statistically significant differences were observed between all intraoral and all mask samples (*p* = 0.021).

When analyzing the individual treatment modalities, statistically significant differences were found on masks between filling therapy (*p* = 0.03), periodontal therapy (*p* = 0.004), and endodontic therapy (*p* = 0.01). However, no statistically significant difference was observed when comparing intraoral samples to mask samples for professional tooth cleaning (*p* = 0.1).

Moreover, the comparison of the detection frequency of different bacteria between treatment modalities did not yield statistically significant results for either the intraoral samples (*p* = 0.9) or the mask samples (*p* = 0.9).

## Discussion

To our best knowledge, this is the first study to compare the intraoral bacteria of the patient before aerosol-producing dental treatments with those on the practitioner’s mask afterwards. Since this is the first study to investigate the origin of microorganisms on the mask, results cannot be directly compared or discussed with literature.

The bacterial species found are consistent with those described in literature for contamination of PPE [6–9, 11, 18].

Moreover, the bacteria detected in the intraoral samples correspond to the commonly described bacteria for the oral cavity [19–23].

The bacteria detected in this study belonged to the natural microbiota of the oral cavity in humans. This is in line with the fact that only healthy patients were included in the study and patients with any risk of infection were excluded. In addition, dental staff adhere strictly to hygiene standards. In the real world, it is therefore possible that obligate pathogens are also part of the spectrum of bacteria that are transmitted and thus pose a significant risk for practitioners and others. Summarized, transmission of pathogenic bacteria cannot be ruled out.

However, even when investigating only healthy individuals, some facultative pathogens were found and should be discussed in more detail.

*Staphylococcus aureus is* a potentially high-risk, sometimes multiresistant, nosocomial pathogen causing a wide variety of diseases from bacteremia to skin abscesses, bone infection, pneumonia, respiratory tract infections, prosthetic joint, surgical site and cardiovascular infections [24]. Attachments to medical implants and host tissues, furthermore formation of mature biofilms contribute significantly to the persistence of chronic infections [25]. Although *S. aureus* was found three times more frequently in oral samples than on the mask, it has a high risk for transmission and infection.

*Staphylococcus epidermidis* was most often detected in all samples and in particular on masks. Like other coagulase-negative staphylococci, it is a facultative and often multiresistant pathogen causing a wide variety of infections in connection with medical and surgical procedures [26, 27]. As part of the commensal microbiome, the bacteria are harmless to healthy individuals, but immunosuppressed or immunocompromised patients may be at risk [28–31]. Same applies for *Streptococcus oralis*, *Staphylococcus capitis, M. luteus* or *R. dentocariosa*, also often detected.

Overall, only a few oral microorganisms could be detected on the mask and the intraoral samples at the same time. This is due to the fact that many species cannot be cultivated, moreover anaerobic bacteria must be excluded from the outset. To cultivate more fastidious bacteria, special culture media would have had to be used.

Many microorganisms were detected in the mouth, but not on the surgical mask, indicating that only a small subset of bacteria were transferred. This is in line with the fact that in most sample pairs only one species matched between the oral cavity and the mask. However, the bacterial load of the oral cavity might be underestimated, since only one quadrant was swabbed during the intraoral sampling but treatment covers the entire oral cavity.

Since many genera have a similar appearance and are difficult to distinguish visually, they may not always have been interpreted correctly, which in turn would explain a low number of matching species. Exemplary are Streptococcus vestibularis and Streptococcus mitis: both viridans streptococci and both looking very similar. To reduce this problem, more than one colony with the same morphology could have been identified. However, given the fact that this is a proof of principle study, the additional effort seems to be of little benefit.

Sampling may also have reduced the microbial diversity and viable bacteria on masks, since the entire exterior surface can never be brought into contact with the agar plate, but rather a central section.

In addition, the MALDI-TOF MS analysis refers to colonies identified as different phenotypes, which may underestimate the bacterial spectrum. In summary, there are probably more viable bacteria on the mask originating from the oral cavity than described in the present study.

An investigation by means of polymerase chain reaction (PCR) would not have been an alternative. In the end, only viable bacteria are of interest as only these can be transmitted representing a potential risk of transmission and infection. Furthermore, a lot of sequencing or species-specific polymerase chain reactions would have been necessary for the investigation. A methodologically enormous additional effort without advantages. So far, there are no other microbiological methods superior to the one chosen. With regard to the scientific question, the chosen method with all its advantages and disadvantages is the most sensible one. There is no method left to better differentiate the colonies.

Although the difference in detection frequency was statistically significant overall and for filling therapy, periodontal therapy and endodontic therapy, this did not apply to professional tooth cleaning. In other words, the composition of microorganisms present in the oral cavity was reflected on the mask during professional tooth cleaning, indicating the highest potential for contamination in this procedure. This finding is supported by the matching species obtained. Interestingly, when comparing the bacterial composition between intraoral samples and mask samples, no statistically significant difference was observed. This is noteworthy considering that the intraoral samples exhibited remarkable similarity despite the inclusion of different patients with diverse treatment requirements, ranging from preventive measures and treatment of periodontitis and dental caries to invasive endodontic therapy.

When using personal protective equipment during treatment, it should always be kept in mind that potentially pathogenic microorganisms may contaminate the mask, becoming a source of contamination for the environment itself [11]. The patient’s health status is sometimes unknown and risk factors for the shift of an at present apathogenic microorganism into a pathogenic one (in case of facultative pathogens) is not always apparent. Finally, colonization of a patient with obligate or facultative pathogens cannot be conclusively clarified. Transmission to vulnerable patients, dentists or assistants is possible. Frequency of exposure and virulence of pathogens determine transmission, infection, and clinical manifestation of diseases frequency [32]. Therefore, it is essential following the recommendations for prevention of nosocomial infections [33, 34] including masks [11]. In addition, a detailed medical history of the patient should always be taken in order to better assess possible infection-related risks.

## Conclusion

In conclusion, the presented study pointed out that oral microorganisms, only originating from the patient’s oral cavity, can be found on the exterior surface of the surgical mask. Thus, the route of transmission could be clearly demonstrated. Future studies should investigate the proportion of contamination from other possible sources such as contaminated water pipes and general air contamination. In addition, a similar study design might be used when investigating the role of viruses.

### Supplementary information

Supplementary Table 1

Supplementary Table Caption

## Data Availability

The dataset used and analyzed during the current study is available from the corresponding author upon reasonable request.
