# Supplementary material for: Detection of viable oral bacteria of the patient on the surgical mask of dentists
Source: BDJ Open. 2024 Jan 16;10:4. doi: 10.1038/s41405-023-00182-4 (PMC10791691; doi:10.1038/s41405-023-00182-4)
Supplement: Supplementary file 2 — Supplementary Table Caption [file 41405_2023_182_MOESM2_ESM.docx]

Supplementary Table 1: Species of microorganisms found in this study; frequency of their detection in intraoral samples, on the mask and according to treatment modality (filling therapy, professional tooth cleaning, periodontal treatment, endodontic treatment). Furthermore, their typical habitat is indicated.
